# Intermediate electrostatic field for the elongation method

**DOI:** 10.1007/s00894-014-2277-6

**Published:** 2014-05-31

**Authors:** Piotr Kuźniarowicz, Kai Liu, Yuriko Aoki, Feng Long Gu, Anna Stachowicz, Jacek Korchowiec

**Affiliations:** 1 Department of Molecular and Material Sciences, Interdisciplinary Graduate School of Engineering Sciences, Kyushu University, 6-1 Kasuga-Park, Fukuoka, 816-8580 Japan; 2 Department of Material Sciences, Faculty of Engineering Sciences, Kyushu University, Kasuga, Fukuoka, 816-8580 Japan; 3 MOE Key Laboratory of Theoretical Chemistry of Environment; School of Chemistry and Environment, South China Normal University, Guangzhou, 510631 China; 4 K. Gumiński Department of Theoretical Chemistry, Faculty of Chemistry, Jagiellonian University, Ingardena 3, 30-060 Krakow, Poland

**Keywords:** Charge sensitivity analysis, Electronegativity equalization equations, Elongation cutoff method, Elongation method, Population analyses, Order-N methods

## Abstract

A simple way to improve the accuracy of the fragmentation methods is proposed. The formalism was applied to the elongation (ELG) method at restricted open-shell Hartree-Fock (ROHF) level of theory. The α-helix conformer of polyglycine was taken as a model system. The modified ELG method includes a simplified electrostatic field resulting from point-charge distribution of the system’s environment. In this way the long-distance polarization is approximately taken into account. The field attenuates during the ELG process to eventually disappear when the final structure is reached. The point-charge distributions for each ELG step are obtained from charge sensitivity analysis (CSA) in force-field atoms resolution. The presence of the intermediate field improves the accuracy of ELG calculations. The errors in total energy and its kinetic and potential contributions are reduced by at least one-order of magnitude. In addition the SCF convergence of ROHF scheme is improved.

## Introduction

Development of linear scaling methods, or order-N methods, is of primary importance in quantum chemistry. There are two main approaches leading to linear scaling. They can be classified as numerical and chemical approaches. The former linearizes every step of Hartree-Fock (HF) or Kohn-Sham (KS) schemes. Nowadays, the construction of HF and KS matrices is linear [1–8]. To reach this goal and to solve HF/KS equations several techniques are applied, for example, prescreening techniques [9], the continuous fast multipole method [1, 10–14], order-*N* exchange [15], near-field exchange [16], tree-code approaches [14, 17], density fitting, density matrix minimization techniques [18–21], linear scaling quadrature methods [22, 23], sparse matrix algebra [24, 25], code parallelization [26], and GPU accelerators [27]. The chemical approaches are based on the so called nearsightedness approximation [28]. It assumes that the electronic structure of a given molecular fragment is predominantly determined by its nearest neighborhood and the influence of very distant fragments is negligible. The nearsightedness approximation is strongly related to generalized product function of McWeeny [29]. Several techniques using generalized product function have been proposed. They are called the fragmentation methods. They differ in conditions imposed on mutual interactions among fragments. In the most advanced fragment molecular orbital (FMO) method [30–34] and elongation (ELG) method [35–41] all interactions are rigorously taken into account. The opposite situation appears when fragments are treated completely independently [42, 43]. Intermediate situations are also possible [44–51]. However, one should remember that by simplifying the interactions and narrowing the variation space a systematic error is introduced.

In this paper we describe a simple way to improve the accuracy of the fragmentation methods. The self consistent field (SCF) calculations for selected molecular fragments are performed in the electrostatic field created by the point-charge distribution of the fragment’s complementary part. The formalism is applied to elongation method where it is an intermediate construction. The field diminishes in each step of the elongation procedure and finally disappears after the electronic structure of the whole system is obtained. This intermediate electrostatic field introduces the long-distance polarization into ELG calculations and is a step beyond the nearsightedness approximation. There have been some attempts to include the polarization effects in charged polymers, however, they limited the fragment’s complementary part to charged groups only. Here, we propose a uniform treatment of the fragment’s complementary part. Point charge distribution was also used by Exner and Mezey [45] to diminish the distance criterion in the adjustable density matrix assembler method.

The paper is organized as follows. First, we briefly describe the ELG methods and charge sensitivity analysis (CSA) used to derive the point-charge distributions. Next, we give information connected with computational details. Then, the performance of the method is shown. The analysis is focused on the accuracy. Finally, the conclusions are given along with the future prospects.

## Elongation and elongation cutoff technique

Elongation method mimics the polymerization/copolymerization reaction mechanism [35, 36]. The electronic structure of the whole molecular system (*M*) is synthesized by enlarging a so called starting cluster (*M*_1_). The SCF calculations performed on *M*_1_ initialize the ELG process. This step is complemented with molecular orbital (MO) localization procedure. The canonical M two fragments: *A*_1_ and *B*_1_ [37]. Next, the size of the system is enlarged by adding a new molecular fragment *C*_1_. The localized molecular orbitals (LMOs) assigned to fragment *A*_1_, which is far away from the chain propagation center, are frozen. The LMOs of *B*_1_ and guess MOs of *C*_1_ constitute the variation space *S*_1_. Then, SCF calculations on *S*_1_ are performed. Such propagation procedure is continued, step by step, until *M* is built. Every SCF step of chain propagation is followed by molecular orbital localization. Canonical MOs are localized onto *A*_i_ and *B*_i_ fragments. The LMOs assigned to *A*_i_ are excluded from variation space (they are frozen in the ELG process). The LMOs assigned to *B*_i_ together with MOs of *C*_i_ constitute the active space (*S*_i_). The whole ELG procedure can be summarized as follows:

1a $$ {M}_1\overset{\mathrm{LMO}}{=}\left({A}_1\Big|{B}_1\right) $$

1b $$ \left\{\begin{array}{l}{M}_2=\left({A}_1\Big|{B}_1+{C}_1\right)\overset{\mathrm{SCF}}{\equiv}\left({A}_1\Big|{S}_1\right)\overset{\mathrm{LMO}}{=}\left({A}_1+{A}_2\right|{B}_2\left)\equiv \right({A}^2\left|{B}_2\right)\\ {}{M}_3=\left({A}^2\Big|{B}_2+{C}_2\right)\overset{\mathrm{SCF}}{\equiv}\left({A}^2\Big|{S}_2\right)\overset{\mathrm{LMO}}{=}\left({A}^2+{A}_3\right|{B}_3\left)\equiv \right({A}^3\left|{B}_3\right)\\ {}\kern2em \vdots \\ {}{M}_{n-1}=\left({A}^{n-2}\Big|{B}_{n-2}+{C}_{n-2}\right)\overset{\mathrm{SCF}}{\equiv}\left({A}^{n-2}\Big|{S}_{n-2}\right)\overset{\mathrm{LMO}}{=}\left({A}^{n-2}+{A}_{n-1}\right|{B}_{n-1}\left)\equiv \right({A}^{n-1}\left|{B}_{n-1}\right)\end{array}\right. $$

1c $$ M={M}_n=\left({A}^{n-1}\Big|{B}_{n-1}+{C}_{n-1}\right)\overset{\mathrm{SCF}}{\equiv}\left({A}^{n-1}\Big|{S}_{n-1}\right) $$

Equations 1a, 1b, and 1c correspond to initialization, chain propagation, and chain termination, respectively. Notice that there is no need to localize MO in the final step (Eq. 1c).

In the ELG scheme the size of variation space is almost constant. One can eventually take advantage of the sparseness in the LMO representation [39, 40, 52, 53]. In the limit of perfect localization, LMOs assigned to the frozen fragment (${\mathbf{C}}_{A^i}$) have no tails in the active fragment and vice versa, LMOs assigned to the active fragment (${\mathbf{C}}_{S_i}$) have no tails in the frozen one. Therefore, instead of constructing the Fock or Kohn-Sham matrix of M_i-1_ (${\mathbf{F}}_{M_{i-1}{M}_{i-1}}^{\mathrm{AO}}$), the ${\mathbf{F}}_{S_i{S}_i}^{\mathrm{AO}}$ block is built. Such elongation cutoff scheme (ELG/C) operates within a low-dimensional subspace,

2 $$ \begin{array}{l}{\boldsymbol{C}}_{S_i}^{\dagger }{\boldsymbol{F}}_{i+1}{\boldsymbol{C}}_{S_i}=\kern1em \left(\begin{array}{cc}\hfill {\mathbf{0}}_{A^i{S}_i}^{{}^{\dagger }}\hfill & \hfill {\mathbf{C}}_{S_i{S}_i}^{\dagger}\hfill \end{array}\right)\kern0.5em \left(\begin{array}{cc}\hfill {\mathbf{F}}_{A^i{A}^i}^{\mathrm{AO}}\hfill & \hfill {\mathbf{F}}_{A^i{S}_i}^{\mathrm{AO}}\hfill \\ {}\hfill {\mathbf{F}}_{S_i{A}^i}^{\mathrm{AO}}\hfill & \hfill {\mathbf{F}}_{S_i{S}_i}^{\mathrm{AO}}\hfill \end{array}\right)\kern0.5em \left(\begin{array}{c}\hfill {\mathbf{0}}_{A^i{S}_i}\hfill \\ {}\hfill {\mathbf{C}}_{S_i{S}_i}\hfill \end{array}\right)\\ {}\kern5em \equiv {\mathbf{F}}_{S_i{S}_i}^{\mathrm{MO}}={\mathbf{C}}_{S_i{S}_i}^{\dagger }{\mathbf{F}}_{S_i{S}_i}^{\mathrm{AO}}{\mathbf{C}}_{S_i{S}_i}\end{array} $$

and avoids the known bottleneck of the SCF calculations, i.e., diagonalization. The lower indices in Eq. (2) are introduced to indicate dimensions and the block ${\mathbf{0}}_{A^i{S}_i}$ is filled with zeros. In addition, the number of two-electron repulsion integrals is substantially reduced as long as the total energy of *M*_i_ is not needed. It was demonstrated that ELG/C scheme is linear in CPU time at HF [52] and KS [53] levels of theory for a linear or quasi-linear polymer. The ELG scheme can be generalized to three-dimensional (3D) systems. In such a generalized ELG scheme the frozen LMOs of a given fragment can reenter the variation space [54–56].

In the ELG (ELG/C) scheme the molecular fragments are not treated equivalently. Namely, the starting cluster does not know its “future” while *S*_n-1_ possesses the whole knowledge of *M*. Such way of building *M* is consistent with the nearsightedness approximation [28] which is a common assumption in the fragmentation methods. However, it is also a source of errors. To reduce this error, the ELG (ELG/C) process can be performed in an approximate field arising from complementary part of *M*_i_, for example its point charge distribution. The ith subsystem’s complementary part is denoted by $\overline{M_i}$. By enlarging the system size (*i* → *n*) the complementary part becomes smaller and finally (*i* = *n*) disappears ($\overline{M_n}$ is a zero set). This electrostatic field is an intermediate construction, therefore, the final energy of *M* should be greater than the reference HF (KS) one.

## Charge sensitivity analysis in force-field atoms resolution

The intermediate point-charge distribution can be easily obtained from charge sensitivity analysis [57]. CSA is based on second-order Taylor expansion of the system’s energy *E*_*M*_ with respect to atomic charges. The CSA formalism in global resolution (without constraints on charge flow) can be summarized in a single matrix equation [58]:

3 $$ \left(\begin{array}{cc}\hfill 0\hfill & \hfill \begin{array}{cccc}\hfill 1\kern1em \hfill & \hfill 1\kern1em \hfill & \hfill \cdots \kern0.62em \hfill & \hfill 1\hfill \end{array}\hfill \\ {}\hfill \begin{array}{c}\hfill 1\hfill \\ {}\hfill 1\hfill \\ {}\hfill \vdots \hfill \\ {}\hfill 1\hfill \end{array}\hfill & \hfill \begin{array}{cccc}\hfill {\eta}_{11}\hfill & \hfill {\eta}_{12}\hfill & \hfill \cdots \hfill & \hfill {\eta}_{1N}\hfill \\ {}\hfill {\eta}_{21}\hfill & \hfill {\eta}_{22}\hfill & \hfill \cdots \hfill & \hfill {\eta}_{2N}\hfill \\ {}\hfill \vdots \hfill & \hfill \vdots \hfill & \hfill \ddots \hfill & \hfill \vdots \hfill \\ {}\hfill {\eta}_{N1}\hfill & \hfill {\eta}_{N2}\hfill & \hfill \cdots \hfill & \hfill {\eta}_{NN}\hfill \end{array}\hfill \end{array}\right)\kern0.5em \left(\begin{array}{c}\hfill -\chi \hfill \\ {}\hfill \begin{array}{c}\hfill {q}_1\hfill \\ {}\hfill {q}_2\hfill \\ {}\hfill \vdots \hfill \\ {}\hfill {q}_N\hfill \end{array}\hfill \end{array}\right)=\left(\begin{array}{c}\hfill q\hfill \\ {}\hfill \begin{array}{c}\hfill -{\chi}_1^{*}\hfill \\ {}\hfill -{\chi}_2^{*}\hfill \\ {}\hfill \vdots \hfill \\ {}\hfill -{\chi}_N^{*}\hfill \end{array}\hfill \end{array}\right) $$

where **η** = {*η*_*ij*_ = ∂^2^ *E*_*M*_/∂*q*_*i*_∂*q*_*j*_} is the hardness matrix, *q* is the total charge and *χ* = ∂*E*_*M*_/∂*q* is the global electronegativity [59, 60] of a molecular system *M* composed of *N* atoms. Vectors **q** = (*q*_1_, *q*_2_, … *q*_*N*_)^T^ and **χ** = (*χ*_1_^*^, *χ*_2_^*^, … *χ*_N_^*^)^T^ group the atomic charges and electronegativities, respectively. The first equation in (3) is a closure relation:

4 $$ q={\displaystyle \sum_{i=1}^N{q}_i} $$

The remaining equations

5 $$ \chi ={\chi}_i={\chi}_i^{*}+{\displaystyle \sum_{j=1}^N{\eta}_{ij}{q}_j},\kern1em j=1,\kern0.5em 2,\kern1em ,\kern0.5em N $$

are the electronegativity equalization equations (*χ*_1_ = *χ*_2_ = … = *χ*_*N*_ = *χ*) [61]. The charge distribution inside M can be obtained by inverting Eq. (3):

6 $$ \kern0.5em \left(\begin{array}{c}\hfill -\chi \hfill \\ {}\hfill \begin{array}{c}\hfill {q}_1\hfill \\ {}\hfill {q}_2\hfill \\ {}\hfill \vdots \hfill \\ {}\hfill {q}_N\hfill \end{array}\hfill \end{array}\right)=\left(\begin{array}{cc}\hfill -\eta \hfill & \hfill \begin{array}{cccc}\hfill {f}_1\kern1em \hfill & \hfill {f}_2\hfill & \hfill \kern1.5em \cdots \hfill & \hfill {f}_N\hfill \end{array}\hfill \\ {}\hfill \begin{array}{c}\hfill {f}_1\hfill \\ {}\hfill {f}_2\hfill \\ {}\hfill \vdots \hfill \\ {}\hfill {f}_N\hfill \end{array}\hfill & \hfill \begin{array}{cccc}\hfill -{\beta}_{11}\hfill & \hfill -{\beta}_{12}\hfill & \hfill \cdots \hfill & \hfill -{\beta}_{1N}\hfill \\ {}\hfill -{\beta}_{21}\hfill & \hfill -{\beta}_{22}\hfill & \hfill \cdots \hfill & \hfill -{\beta}_{2N}\hfill \\ {}\hfill \vdots \hfill & \hfill \vdots \hfill & \hfill \ddots \hfill & \hfill \vdots \hfill \\ {}\hfill -{\beta}_{N1}\hfill & \hfill -{\beta}_{N2}\hfill & \hfill \cdots \hfill & \hfill -{\beta}_{NN}\hfill \end{array}\hfill \end{array}\right)\left(\begin{array}{c}\hfill q\hfill \\ {}\hfill \begin{array}{c}\hfill -{\chi}_1^{*}\hfill \\ {}\hfill -{\chi}_2^{*}\hfill \\ {}\hfill \vdots \hfill \\ {}\hfill -{\chi}_N^{*}\hfill \end{array}\hfill \end{array}\right) $$

Here, **β** = {*β*_*ij*_ = ∂^2^ *E*_*M*_/∂*v*_*i*_∂*v*_*j*_ = − ∂*q*_*j*_/∂*v*_*i*_ = − ∂*q*_*i*_/∂*v*_*j*_} is the polarization matrix (linear response matrix). The vector **v** = (*v*_1_, *v*_2_, … *v*_*N*_) denotes the external potential due to nuclei. The remaining undefined quantities are the global hardness [62, 63] *η* = ∂^2^ *E*_*M*_/∂*q* ^2^ and the Fukui function (FF) [63] vector **f** = {*f*_*i*_ = (∂*q*_*i*_/∂*q*)_*v*_ = − (∂*μ*/∂*v*_*i*_)_*q*_}. The hardness matrix **η** and the vector of atomic electronegativities **χ** are the base parameters of CSA. In the force-field atoms resolution they depend on the atomic number, hybridization and local chemical environment of atoms constituting the system. We have recently derived these parameters for different population analyses [64, 65].

## Computational details

All ELG (ELG/C) calculations were performed at restricted open-shell Hartree-Fock (ROHF) level of theory using GAMESS package [66, 67]. Three different basis sets, namely: STO-3G, 6-31G, and 6-31G(d), were applied. The alpha-helix conformer of polyglycine was taken as a model system. The starting cluster was built of 15 amino acid units. In each step of the elongation eight units were frozen and another eight units were added to the system. This ELG propagation scheme is denoted as 15/8. The elongation process was terminated for 55 glycine units. In the case of ELG/C calculations, the cutoff procedure was initialized for a system made up of 31 units (15/8:31). The geometry of alpha-helix was the same as in our earlier paper [52]. We have chosen this conformer since, for a given partitioning scheme, the error in its total energy was bigger than for other conformers (C5, C7, and 3_10_-helix).

The point charge distribution for different population analyses was obtained from CSA calculations. Five different charge distributions were used, namely, Bader (B) [68], Hirshfeld (H) [69], Mülliken (M) [70], Natural (N) [71], and Voronoi (V) [72] population analyses. The ELG calculations were carried out for each charge distribution. The errors in total (tot), kinetic (kin), and potential (pot) energies were defined with respect to the conventional (supermolecule) ROHF energies:

7 $$ \varDelta {E}_x={E}_x^{\mathrm{ELG}}-{E}_x^{\mathrm{ROHF}},\kern1.75em x=\left(\mathrm{tot},\mathrm{kin},\mathrm{pot}\right) $$

By definition *ΔE*_*M*_ = *ΔE*_tot_ is greater than zero since ELG (ELG/C) is a variational method. There are no such restrictions on its components, therefore, *ΔE*_kin_ (*ΔE*_pot_) may be either negative or positive. We have chosen ROHF calculations since the propagation at RHF level of theory would require saturation of “broken” bonds. This means that each intermediate subsystem (15, 23, 31, 39, and 47 units) would have to be saturated by a hydrogen atom. To avoid perturbation created by this artificial hydrogen atom ROHF scheme was selected. One can expect that the intermediate electrostatic field should stabilize the system and should improve the SCF convergence. The proposed modification of ELG (ELG/C) scheme has no influence on CPU time since one-electron integrals are computed only once, before the SCF process.

## Results and discussion

**Fig. 1:**
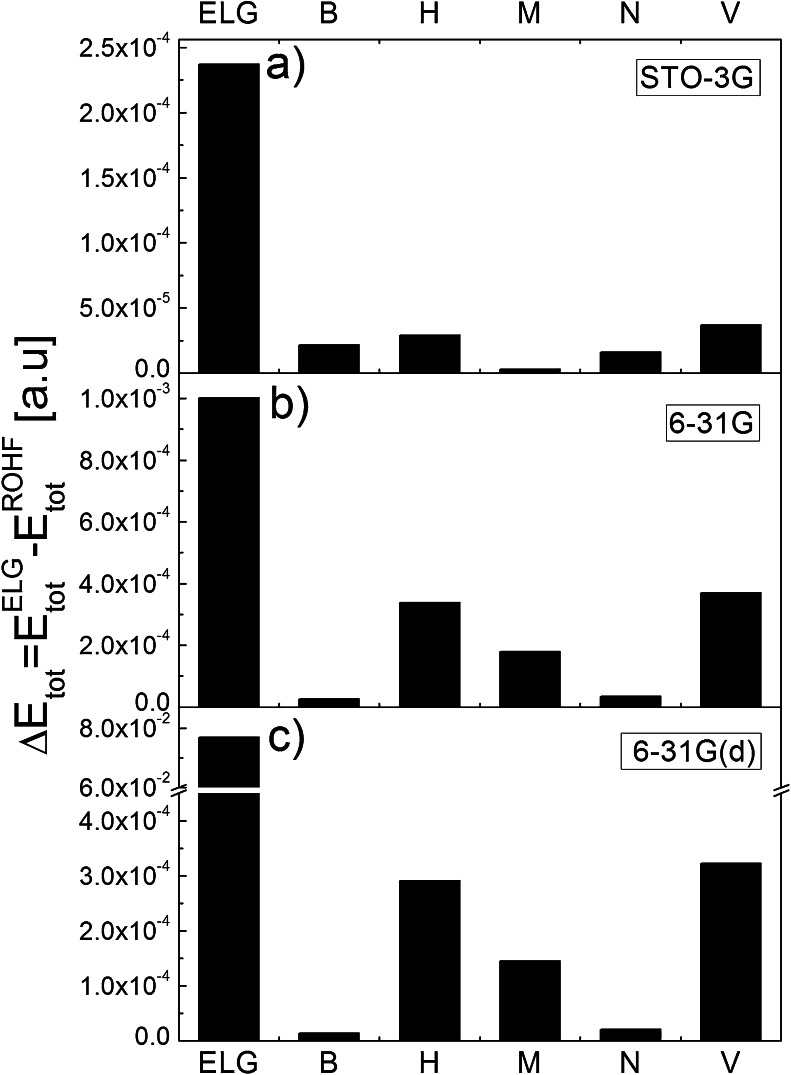
The error [a.u.] in the total energies introduced by 15/8 ELG and modified ELG schemes [with Bader (B), Hirshfeld (H), Mülliken (M), Natural (N), and Voronoi (V) charge distributions] with respect to conventional ROHF calculations for polyglycine chain made up of 55 units. Parts (**a**), (**b**), and (**c**) correspond to STO-3G, 6-31G, and 6-31G(d) basis sets

The errors introduced by ELG and modified ELG schemes are plotted in Fig. 1. Parts a, b, and c correspond to STO-3G, 6-31G, and 6-31G(d) basis sets, respectively. The error in the total energy is always positive. The ELG and modified ELG methods are variational, therefore the calculated energies are higher than the reference ROHF energies. All energies are computed for the final system built up of 55 units. The intermediate energies cannot be directly compared with the reference ROHF energies since they have quite a different meaning. The first error bar in each figure corresponds to standard ELG calculations with ${\mathbf{q}}_{\overline{M_i}}=0$ (no field). The remaining error bars correspond to different population analyses employed in the modified ELG procedure. The error in ELG calculation depends on the basis set and increases with its size. For 6-31G(d) basis set it is about two-orders of magnitude greater than for the minimal basis set. The presence of the intermediate field arising from charges from every population analysis reduces the error in the total energy. The reproduction of conventional ROHF energies is improved by about one order of magnitude for STO-3G (Fig. 1a) and 6-31G (Fig. 1b) basis sets. The improvement for 6-31G(d) basis is the most pronounced (Fig. 1c). The error is reduced by three orders of magnitude for B, M, and N population analyses. For all basis sets the modified ELG scheme with V and H charges works slightly worse than with B, M, and N charges. The changeability in total energy with the basis set size is the smallest for B and N charges. The errors in total energies are equal to 0.01, 0.02, and 0.02 kcal mol^-1^ for STO, 6-31G, and 6-31G(d) basis sets, respectively.

Therefore, by taking the long-distance polarization into account, even in the simplified way, the reproduction of the reference ROHF energy is much better and the error does not exceed 1 kcal mol^-1^. It should also be mentioned that, although Fig. 1 corresponds to ELG and modified ELG schemes, they present the errors in the ELG/C and modified ELG/C schemes. The reason for that is that the cutoff error (*E*_tot_^ELG^ − *E*_*t*ot_^ELG/C^) is at least two orders of magnitude smaller than the elongation error (*E*_tot_^ELG^ − *E*_tot_^ROHF^).

**Fig. 2:**
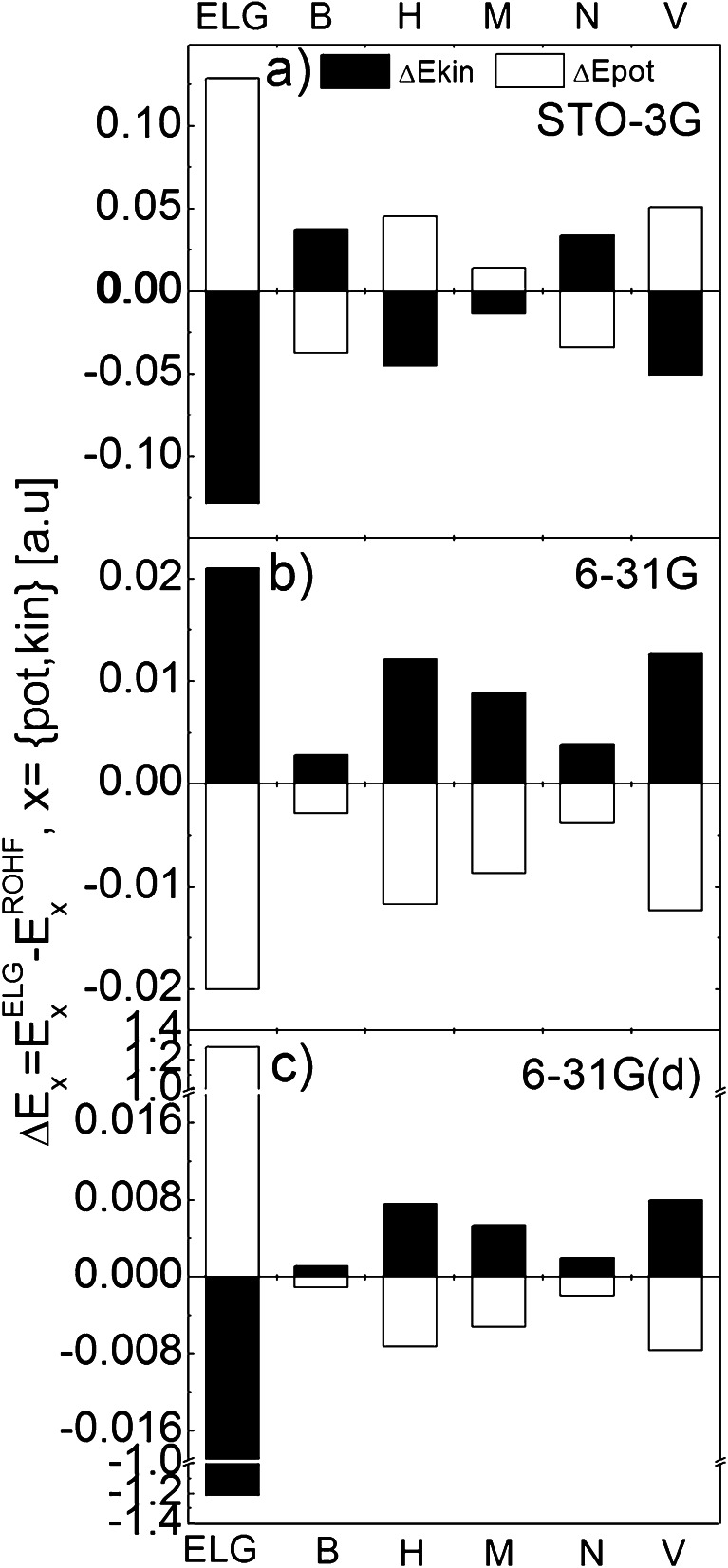
The error [a.u.] in the potential (white bars) and kinetic (black bars) energies introduced by 15/8 ELG and modified ELG schemes [with Bader (B), Hirshfeld (H), Mülliken (M), Natural (N), and Voronoi (V) charge distribution] with respect to conventional ROHF calculations for polyglycine chain made up of 55 units. Parts (**a**), (**b**), and (**c**) correspond to STO-3G, 6-31G, and 6-31G(d) basis sets

To understand the improvement in reproduction of the system’s total energy we have decomposed *E*_tot_^ELG^ into its potential (*E*_pot_^ELG^) and kinetic (*E*_kin_^ELG^) components. The errors with respect to conventional potential (*E*_pot_^ROHF^) and kinetic (*E*_kin_^ROHF^) energies are illustrated in Fig. 2. The black error bars correspond to *ΔE*_kin_ while white error bars to *ΔE*_pot_. Again, the first error bar in each figure corresponds to standard ELG scheme. As it was mentioned in Computational details, the errors in potential and kinetic energies may be either positive or negative. It is clearly seen in Fig. 2a. Depending on the population analysis, the error in kinetic energy is positive (B and N) or negative (H, M and V). Regardless of the basis set and the population analysis, the error in potential energy always has the opposite sign to *ΔE*_kin_. Its magnitude is almost the same as that of *ΔE*_pot_. This error cancelation causes the accuracy in the total energy to be one order of magnitude greater than in its potential and kinetic components. Based on the virial theorem, a different ratio *ΔE*_pot_/*ΔE*_kin_ should be expected. However, one should remember that the virial theorem is exact for the true ground-state wave function. The approximate wave function fulfils it only approximately. For more extended basis sets [6-31G and 6-31G(d)], *ΔE*_pot_ for modified ELG scheme is negative and *ΔE*_kin_ is positive. In the case of standard ELG scheme, *ΔE*_pot_ is negative for 6-31G and positive for 6-31G(d) basis sets, respectively.

It is clear from the figures that the intermediate electrostatic field improves the reproduction of kinetic and potential energies. Such improvement is evident for 6-31G(d) basis set. One should expect such behavior since slightly populated polarization functions on heavy atoms should be sensitive to the intermediate field used in the modified ELG calculations. All the results of modified ELG calculations at 6-31G(d) basis set show that the kinetic energy is overestimated due to the influence of point charges.

**Fig. 3:**
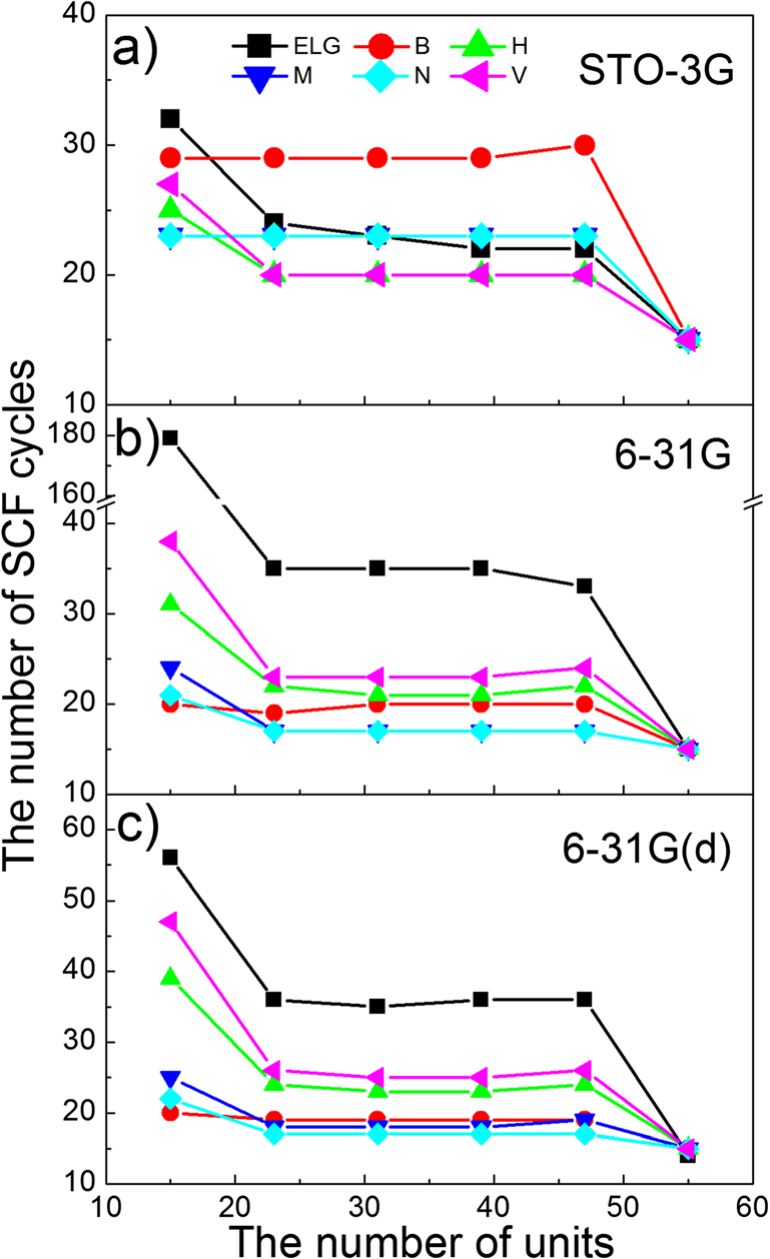
The SCF convergence during ELG and the modified ELG [with Bader (B), Hirshfeld (H), Mülliken (M), Natural (N), and Voronoi (V) charge distribution] calculations. Parts (**a**), (**b**), and (**c**) correspond to STO-3G, 6-31G, and 6-31G(d) basis sets

Finally, let us analyze the SCF convergence during ELG calculations. All intermediate subsystems are radicals (dublets). Only the whole system is closed-shell. The SCF convergence for radicals is worse than for closed-shell singlet states. The SCF convergence is illustrated in Fig. 3. Parts a, b, and c correspond to STO-3G, 6-31G, and 6-31G(d) bases, respectively. One can observe that, except for B, M, and N charges at STO-3G basis set calculations, the field stabilizes the radicals. It means that fewer SCF cycles are required to reach the same assumed accuracy in comparison to standard ELG scheme. The differences between ELG and modified ELG schemes are more pronounced for the starting cluster. For larger basis set, the M and N population analyses give the fastest convergence. In general, the natural population analysis (N) gives the best performance of calculations both in accuracy and convergence. At the end of the elongation process the number of iterations is the same for all curves. The system is then closed-shell and the number of iterations in the SCF cycle is small.

## Conclusions

A simple modification of the elongation (elongation cutoff) method was proposed. Namely, the intermediate electrostatic field was introduced. The field is exerted by distributed monopoles located in the positions of atoms of the system’s complementary part and it disappears in the final stage of the elongation calculations. Therefore, it does not violate the variational character of the ELG method. The modified ELG scheme was tested for alpha-helix of polyglycine chain (55 glycine units). Several population analyses were applied. Charges were computed using CSA scheme, independently of the quantum chemical calculations. It was shown that the long-distance polarization, introduced by the field, improved the performance of the ELG method. The errors in the total, kinetic, and potential energies were reduced by at least one order of magnitude. The natural, Bader, and Mülliken population analyses gave the best agreement with the reference, conventional ROHF energies for the largest basis set. The proposed method can be easily adopted to other fragmentation techniques.

The modified ELG method improved the convergence of the SCF process at ROHF level of theory. The temporary electrostatic field stabilized the intermediate radical, therefore fewer cycles were involved during the SCF step. We plan to adopt the formalism at RHF and UHF levels of theory.
